# Recognizing and mediating bureaucratic barriers: increasing access to care through small private providers in Kenya

**DOI:** 10.12688/gatesopenres.13313.1

**Published:** 2021-06-17

**Authors:** Lauren Suchman, Dominic Montagu

**Affiliations:** 1 University of California San Francisco, San Francisco, CA, USA

**Keywords:** Health financing, health insurance, evaluation, policy implementation, private providers, Kenya

## Abstract

**Background: **Equitable access to health services can be constrained in countries where private practitioners make up a large portion of primary care providers, making affordability a challenge. Expanding purchasing arrangements in many countries has helped integrate private providers into government-supported payment schemes and reduced financial barriers to care. However, private providers often must go through an onerous accreditation process to enroll in government-supported financing arrangements. The difficulties of this process can be exacerbated where health policy is changed often and low-level bureaucrats must navigate these shifts at their own discretion, effectively re-interpreting or re-making policy in practice. This paper analyzes one initiative to increase private provider accreditation with social health insurance (SHI) in Kenya by creating an intermediary between providers and SHI officials.

**Methods:** This paper draws on 126 semi-structured interviews about SHI accreditation experience with private providers who were members of a franchise network in Kenya. It also draws on four focus group discussions conducted with franchise representatives who provided accreditation support to the providers and served as liaisons between the franchised providers and local SHI offices. There was a total of 20 participants across all four focus groups.

**Results:** In a regulatory environment where regulations are weak and impermanent, officials created an accreditation process that was inconsistent and opaque: applying rules unevenly, requesting bribes, and minimizing communication with providers. The support provided by the implementing organizations clarified rules, reduced the power of local bureaucrats to apply regulations at their own discretion, gave providers greater confidence in the system, and helped to standardize the accreditation process.

**Conclusions:** We conclude that intermediary organizations can mitigate institutional weaknesses, reduce barriers to effective care expansion caused by street-level bureaucrats, and facilitate the adoption of systems which reduce rent-seeking practices that might otherwise delay or derail initiatives to reach universal health coverage.

## Introduction

The United Nations Sustainable Development Goals (SDG) call for all countries to achieve Universal Health Coverage (UHC) by 2030. This will require expanding both financing and service availability. Many low- and middle-income countries (LMICs) are depending on expanded national budget allocations, and new social health insurance (SHI) schemes to better align health financing with UHC priorities. However, the extent to which these schemes can effectively advance progress toward UHC will depend on governments’ ability to contract with a sufficient number of health care providers to create a pool of quality service delivery options that are geographically and financially accessible. In sub-Saharan Africa, where private facilities provide almost half of the outpatient health services offered ( Chakraborty & Sprockett, 2018; Grépin, 2016), ensuring that private providers are accredited with local SHI systems is vital to achieving UHC. However, many barriers exist: while public facilities are automatically enrolled into SHI schemes, private providers must go through a cumbersome formal accreditation processes ( Sieverding *et al.*, 2018). Identifying the key roadblocks private providers face in the accreditation process and finding ways to ease the burdens of bureaucratic functions is likely to be a key determinant of expanding affordable near-to-patient healthcare services in support of UHC. 

Drawing on interviews with private providers in Kenya, an LMIC country that has recently expanded SHI contracting, this paper analyzes a programmatic effort to increase private provider accreditation by mediating between providers and SHI officials. According to Lipsky, high-level policies often are re-worked on the ground where low-level bureaucrats interact with the public, reinterpreting broad or vague policies to address the immediacy of changing daily circumstances ( Lipsky, 1980). Our study looks at an intervention to reduce the variations in these ‘street-level’ applications of policy by assisting private providers to enroll with the SHI scheme and, as a result, increase the number of healthcare service delivery points available to insured populations. 

### Literature review

Regulations that ought to manage service provision often mistakenly constrict provision instead ( Montagu & Goodman, 2016). The relationship between private providers in LMICs and the government systems that license, regulate, and sometimes contract to them often are minimal and conflicted. Regulations that address private practice are frequently limited and enforcement systems weak ( Batley, 2006). Where such systems are effective at reaching private providers on a regular basis, providers rarely see these systems as beneficial, but rather as an imposition: a *de jure* or *de facto* tax on business. Regulatory quality, enforcement, and *de facto* control of quality were all rated as “very poor” in more than 80% of LMIC countries surveyed by the World Bank ( World Bank Group, 2011).

***Regulatory complexity and unintended consequences.*** The contradiction between law and practice is not unusual, nor limited to LMICs. In many countries regulation of professionals is devolved to professional bodies – the American Medical Association sets standards for medical accreditation in the United States (US); the British Medical Association does the same in the United Kingdom (UK); pharmacists, phlebotomists, dentists, and many other medical specialist organizations do the same across the Organisation for Economic Co-operation and Development (OECD) ( Benton *et al.*, 2017). As is often the case with professional organizations, what begins as self-regulation can easily evolve into something else ( Salamon, 2000). Through monopolistic behaviors, providers and their representative organizations can accelerate a natural tendency inherent to regulatory bureaucracies: growth in the number and complexity of regulations. This is common in all countries and more so in countries with weak governance systems ( Saltman & Busse, 2002). An unintended effect of the widespread devolution of health system regulations in the early 2000s was the creation of more layers of bureaucracy, each empowered to develop its own laws and enforcement systems ( Cobos Muñoz *et al.*, 2017) ( Saltman & Bankauskaite, 2006). At the best of times this increased the regulatory burden on private providers. At the worst, new regulations contradicted existing regulations, with the effect that providers were inevitably doing something forbidden no matter which rule they elected to follow. In such a situation policies multiplied to the extent that confusion existed regarding intent, even when specific regulations were absent ( Davis, 2017).

One result of this kind of regulatory complexity is that all actors in a system are eventually operating outside of the rules of one layer of government or another and are therefore susceptible to incurring a host of new operating costs to become compliant, to engaging in administrative or legal efforts to clarify guidance, or to making informal payments in order to avoid enforcement of rules ( Kisunko *et al.*, 1999). As regulations proliferate, the enforcers of rules, such as low-level bureaucrats, become increasingly powerful and autonomous ( Ramiro *et al.*, 2001). Where multiple unclear and contradictory rules exist, any enforcement decision by a local administrator or program officer can always be justified. The incentives to not participate grow, reducing both formal, and overall, care quality and availability.

A common theme in LMICs is that policies exist, often many of them, but the legislation and regulatory guidance that clarify how policies are to be implemented and enforced are lacking ( Kaufmann, 1997). As Klitgaard has noted, corruption flourishes in the absence of accountability, and complexity makes accountability more difficult ( Klitgaard, 1988), Indeed, studies have shown higher rates of corruption in countries with more tiers of government, especially in LMICs ( Fan *et al.*, 2009). Research has shown that the large information asymmetry between providers and patients, and the complexities of principal-agent problems when payers’ incentives diverge from both providers and clients, all conspire to make healthcare systems particularly susceptible to corruption and inefficiency ( Mostert *et al.*, 2015) ( Vian, 2008). While corruption can exist from the systemic and institutional levels down to the level of the individual, making it notoriously difficult to define ( Johnston, 1996), the street-level bureaucrats who have regular direct contact with the public are unlikely to benefit from institutional schemes ( Miller, 2006). A more significant barrier to system efficiency, we postulate, is that complex regulatory systems create an environment in which low-level bureaucrats are almost unable to play by the rules. In these instances, a lack of consistency and transparency may hinder regulatory processes just as much, if not more than, overt requests for bribes.

***The inevitability of sand in the gears.*** Street-level bureaucracy is particularly potent in complex regulatory systems such as health services. Low-level bureaucrats re-work policy in these situations as a means to cope with daily struggles, such as insufficient resources, and vague or conflicting agency goals. And indeed, the empowered autonomy of local government agents is not always a bad thing: the same systemic regulatory incoherence that enables negative behaviors can also allow local administrators to make decisions and cut through otherwise impenetrable red tape or make their own work goals more predictable and achievable. Even in OECD countries, community health workers must make their own adjustments to formal policy in order to compensate for the shortcomings of the health system writ large ( George, 2008).

In contrast, the policy landscape tends to shift regularly in LMIC settings, creating a complex regulatory environment in which rules overlap and contradict each other. In such an environment, street-level bureaucrats face a different predicament than they do in higher-income countries with more stable governance; regulatory enforcement becomes an exercise not of choosing which rules to ignore or modify, but which to choose when offered a conflicting array of choices. The results are bottlenecks and avoidable costs which reduce supply of care.

Of the research on this barrier to health system functioning in LMICs, some studies point to the importance of flexibility and a minimum level of decision-making power for low-level bureaucrats, which allows them to do their jobs more effectively ( Crook & Ayee, 2006). However, this flexibility left room for cases in which bureaucrats did not understand a rule or how to apply it and were left to interpret vague definitions on their own ( Agyepong *et al.*, 2016). In other cases, allowing street-level bureaucrats to interpret policy on their own could impede program implementation when, for example, bureaucrats implemented a new policy according to their own values and views or to maintain a certain reputation in their community ( Walker & Gilson, 2004) ( Kaler & Watkins, 2001). However, researchers conceded that bureaucrats acting as impediments to program implementation sometimes operated in contexts where rules contradicted each other and budgets had not been allocated directly to program activities ( Kamuzora & Gilson, 2007).

In this paper, we look at an intervention aiming at reducing the barrier to services posed by low-level governmental workers operating without clear rules or guidance.

### The Kenyan context

While public sector providers in Kenya both have their salaries paid by government and work in government-financed facilities, private sector providers have limited interaction with government systems. These providers are expected to be licensed with the Kenya Medical Practitioners and Dentists Council (KMPDC) and to renew this license annually. However, licensing and regulation for the private health sector is fragmented and under-resourced, with one World Bank report referring to the regulatory arena as a “free for all” ( Barnes *et al.*, 2010). Private providers historically have had inconsistent interaction with the government at best and at worst hardly any interaction at all. However, this trend is changing as the National Hospital Insurance Fund (NHIF) continues to expand and opportunities for private providers to come into contact with government regulatory systems increase.

As in other countries where devolution has shifted governmental power dynamics, local administrators are now taxed with supervising licensure and regulatory issues as well as accreditation and payment through the expanded NHIF, giving them significantly more responsibility and authority than they had under the prior centralized system ( McCollum *et al.*, 2018; Obosi, 2019; Suchman, 2018). The motivation for devolution in Kenya has been to reduce corruption and increase responsiveness by bringing government closer to the people. However, constant shifts in policy are now filtered through several new levels of government before reaching providers on the ground. This creates a confusing and unpredictable environment for private primary health clinics.

In 2013, an NGO-led initiative began working to break through this tangle of new bureaucratic complexity.

### The African Health Markets for Equity (AHME) NHIF accreditation assistance intervention

The African Health Markets for Equity (AHME) initiative aimed to increase access to quality, private health care for the poorest populations in Kenya and Ghana. In Kenya, the program ran from 2012–2019 and incorporated social franchising through Marie Stopes Kenya and Population Services International Kenya, and external quality accreditation systems. It also was designed to facilitate NHIF funding for the poor being delivered through private primary care clinics. When AHME started, NHIF contracting in Kenya had only recently expanded to private clinics. Thus, most Kenyan providers were unaccredited and ineligible for reimbursement. In response to the low accreditation numbers, the social franchising partners developed an intervention that involved preparing the AHME-supported providers for NHIF accreditation inspection (including preparing paperwork and obtaining necessary licenses), scheduling the inspection, and following up directly with NHIF officials to ensure that applications were vetted in a timely manner and feedback given to providers when necessary to fill gaps in their application. 

The findings detailed below were developed from the qualitative component of a mixed-methods evaluation of the AHME program. The objectives of this study were to document and analyze participating providers’ experiences with the AHME package of interventions, including the NHIF accreditation assistance intervention.

## Methods

Data for this paper were collected as part of the qualitative evaluation of the African Health Markets for Equity (AHME) program, which was conducted by the University of California San Francisco (UCSF). The AHME intervention package included social franchising enrollment, ( Viswanathan *et al.*, 2016) a quality improvement/quality accreditation initiative (see www.safe-care.org for more information), and access to loans for facility improvement or expansion (see www.medicalcreditfund.org/ for more information). In order to make these quality services more affordable for low-income populations, the AHME partners (Marie Stopes International and Marie Stopes Kenya, Population Services International and Population Services International Kenya, the PharmAccess Foundation, and formerly the International Finance Corporation) worked with the NHIF to identify people living in poverty and enroll them into the NHI scheme for free. The partners then applied the intervention described above to ease the accreditation process for providers so that they could serve low-income patients at an affordable cost. Participating providers included all providers in the AHME-supported franchise networks who wished to pursue NHIF accreditation, but were not yet accredited.

### Sampling

***Providers.*** As shown in Table 1, this analysis draws from a dataset of 126 semi-structured interviews with private providers in Kenya. This includes 24 interviews conducted in 2013, 52 interviews conducted in 2015 and 50 interviews conducted in 2017. This sample size was determined according to the sample size selected for the quantitative component of the mixed-methods evaluation and shifted over time as more clinics were enrolled into the AHME franchising intervention, ultimately representing approximately 50% of all franchised clinics. For the purposes of this study, individual health facilities, not physicians, were considered “providers” and although there was minimal overlap in sampling across rounds of data collection, two facilities were visited more than once (both Rounds 2 and 3). However, because identifying information was not collected for interview participants, we have no way of confirming if the same person from these facilities participated in more than one interview. The qualitative dataset consists of semi-structured interviews with nurses, midwives, doctors, clinical officers, and other key decision-makers at private health facilities that were members of one of the AHME partner social franchises, as well as facilities that had been approached to join the franchise network but declined. In most cases, only one person was interviewed at each facility.

**Table 1.** Provider sample characteristics. NHIF=National Hospital Insurance Fund; AHME=African Health Markets for Equity.

	2013	2015	2017
**Number of** ** interviews**	24	52	50
**Average age**	49 years old	47 years old	41 years old
**Males**	9	31	32
**Females**	15	26	18
**Average years in** ** practice**	21 years	20 years	17 years
**Owners of facility**	75%	74%	68%
**Region**			
Central	0%	0%	18%
Coast	0%	0%	18%
Eastern	71%	32%	12%
Nairobi	29%	37%	16%
Nyanza	0%	0%	28%
Rift Valley	0%	31%	8%
**Provider title**			
Medical Doctor	8%	9%	8%
Nurse	42%	42%	30%
Community Health/ Auxiliary Nurse	17%	9%	7%
Midwife	4%	2%	11%
Clinical Officer	21%	17%	28%
Other/Unknown	8%	21%	16%
**Facility type**			
Clinic	71%	62%	50%
Hospital	17%	9%	16%
Health Center	8%	8%	10%
Maternity Home/ Nursing Home	4%	19%	6%
Pharmacy	0%	2%	8%
**AHME ** **interventions**			
Amua	42%	48%	30%
Tunza	58%	40%	30%
NHIF accreditation	37%	35%	38%
Quality improvement intervention	n/a	38%	56%
Loans and business support	n/a	21%	54%

During each round of data collection, the AHME social franchising partners, Marie Stopes Kenya (MSK) and Population Services International Kenya (PSI Kenya), provided the research team with lists of providers franchised under the Amua (MSK) and Tunza (PSI Kenya) networks. During Rounds Two (2015) and Three (2017) of data collection, the franchise partners also provided lists of providers who had been contacted to join the franchise, but had declined. These clinics were included in the sample to provide a point of comparison against which the research team could better determine the effects of the AHME interventions.

Using the provider lists provided by MSK and PSI Kenya, we used a purposeful criterion sampling strategy ( Palinkas *et al.*, 2015) to design a sample that represented providers with a mix of experiences with the AHME intervention package. In order to capture potential effects of the NHIF accreditation assistance intervention, we also selected facilities based on their NHIF accreditation status in Rounds 2 and 3 (2015 and 2017). Interviews were conducted with providers in a range of facility types across six regions (Nairobi, Eastern, Coast, Central, Rift Valley, Kajiado) during the three rounds of data collection.

All potential participants in a franchise network were made aware of the study by the program implementers (the franchising organizations) and then approached in person by a member of the research team who invited them to join the study. Almost all franchised providers agreed to be interviewed after being approached. The non-franchised providers were approached directly by the research team and invited to participate in an interview. Refusal rates for this population were not available at the time this paper was written. In order to reduce potential bias in the sample we attempted to make it clear that the research team was independent from the program implementing partners when approaching providers. In addition, field staff were trained in qualitative interviewing techniques specifically meant to reduce bias, such as asking open-ended questions and responding to interviewees with neutral expressions.

***Franchise representatives.*** In addition to interviews with private providers, this analysis draws from focus group discussions (FGD) conducted in 2018 with franchise representatives who worked with the AHME-supported providers. These representatives were staff at either MSK or PSI Kenya and acted as liaisons between the providers and NHIF officials, helping providers to prepare for accreditation and then working with the NHIF officials to ensure that these applications moved along quickly and smoothly. Focus groups with franchise representatives were conducted only in 2018 in order to provide context for the AHME qualitative evaluation team as they concluded their analysis. To select FGD participants, the AHME implementing organizations were contacted and asked to provide the names of at least three franchise representatives who would be willing to talk with the qualitative evaluation team with the aim of conducting two FGDs at each organization each with at least three participants. A total of four focus group discussions were conducted (two at each organization) with a total of 20 participants across all four groups. All of the potential participants who were approached agreed to participate.

### Data collection, processing and analysis

***Providers***. The UCSF team partnered with Innovations for Poverty Action (IPA), a research organization based in New Haven, CT with country offices across the globe to collect provider data in Kenya. IPA recruited field interviewers who were then trained by the UCSF team working with IPA staff.

Data collection with providers took approximately one month during each round. Field staff traveled to clinics where providers had already been contacted by IPA and agreed to participate in an interview. However, providers did not have additional information about the interviewer they would be working with ahead of time. Upon arriving at the interview site, interviewers confirmed that the interviewee was one of the key people at the facility who had been involved in decision-making around whether or not to participate in the AHME interventions and obtained informed consent from the providers prior to conducting semi-structured interviews that lasted approximately 60 minutes each. Interviews were most often conducted at the health facility where the provider worked. Where possible, interviews took place in a private consulting room or office at the health facility to ensure privacy and data quality. During each round of data collection, interviewers used a semi-structured interview guide that had been written by the research team at UCSF and piloted by IPA using franchised providers who were eligible for the study. Piloting in each round resulted in small changes to the wording of certain questions, but no substantive changes to the overall guide. Providers were asked about their experiences with the AHME interventions and their knowledge of or desire to join any interventions in which they were not currently participating. In Rounds Two and Three (2015, 2017) of data collection, providers also were asked about their perceptions of and experiences with the NHIF. All guides and consent forms can be found as extended data ( Montagu & Suchman, 2020).

All interviews were recorded using digital recorders in the language the interviewee was most comfortable using. In anticipation that all respondents would not be comfortable conducting a full discussion in English, interview guides were first developed in English and then professionally translated into Swahili to ensure that the translations accurately captured the intended meanings of the original guide. In addition, IPA field staff were all Kenyan and native Swahili speakers. Recordings were translated and transcribed simultaneously by a team of professional Kenyan transcriptionists who had been trained on key terms. IPA research assistants were responsible for back-checking interviews, including ensuring translation accuracy. After the back-checking process was concluded, IPA transferred the transcripts to UCSF for analysis. Transcripts were not returned to participants for comment.

***Franchise representatives.*** Data collection with franchise representatives took place in June 2018 and all of the focus group discussions were conducted by the UCSF team, which consisted of a PhD-level researcher and a program manager, both with experience conducting qualitative interviews and focus groups. Focus group participants were debriefed ahead of time by their supervisors who shared the purpose of the study. The FGDs were held in a private meeting room at either the MSK or PSI Kenya offices. The UCSF team obtained verbal informed consent from all participants before starting discussion, each of which lasted approximately 90 minutes. Discussion topics included the representatives’ daily responsibilities, the nature of their relationships with providers, and details of their work with the NHIF. 

All FGDs were recorded using digital recorders and all were conducted in English, which was familiar to all participants. Recordings were transcribed by a professional Kenyan transcriptionist who had been trained on key terms and were back-checked by UCSF staff. Transcripts were not returned to participants for comment.

All transcripts were coded by two researchers from the UCSF team with assistance from two IPA research assistants during the final round of data analysis with the exception of the FGDs, which were coded solely by one UCSF researcher due to the complexity of coding FGD transcripts. Coders used the popular qualitative analysis program Atlas.ti version 8. Open source alternatives to Atlas.ti include Qualcoder and RQDA. Dedoose is a paid, but lower-cost alternative. The UCSF team used an inductive, thematic approach to coding and analyzing the interviews. This was because there was little existing literature on private providers’ experiences with social health insurance in general and with the Kenyan NHIF specifically from which to derive prior theories.

An initial coding scheme was created in 2013 based on thematic coding of a sub-set of the interviews from each country and each interview was coded using an open coding approach, in which codes were derived from the data. Common codes were identified across the interviews and grouped into code families and sub-codes. Codes aligned with the main themes of the evaluation, specifically provider experiences with each of the AHME interventions, challenges and benefits of the interventions, and provider experiences with NHIF accreditation. During subsequent rounds of analysis, codes were refined to allow for new priorities in analysis while ensuring continuity across rounds. New codes were developed, also inductively, for the single round of franchise representative FGDs.

The coding team reviewed the codebook together during each round of analysis to ensure common understanding of codes and consistency in application. During each round of coding, coders jointly coded 2-3 transcripts and discussed questions and discrepancies to determine inter-coder reliability before beginning independent focused coding. The first author also reviewed a sub-set of coded interviews during each round to check for consistency across coders. The coding process indicated that saturation was reached for themes related to NHIF experience and both preliminary and final findings were shared with the participants and the implementing organizations. The implementing organizations had the opportunity to comment on the preliminary findings and the research team took these comments into account while preparing final documentation while taking care to maintain the integrity of the external evaluation.

### Ethical approvals

Ethical approval for the AHME qualitative evaluation was provided for each round of data collection by the Kenya Medical Research Institute (Protocol #Non SSC no. 411), and with “exempt” status from the Institutional Review Board of UCSF. According to the requirements of the KEMRI IRB, informed verbal consent was obtained from clients in Kenya before interviews were conducted. Providers were given the option to withdraw their participation at any time with no consequences for their participation in the AHME interventions. To thank them for their time, participating providers were given a small gift worth approximately five US dollars, such as a pack of rubber gloves.

## Results

### Building a relationship & reputation with government

Many private providers in Kenya have inconsistent interaction with the government at best. Indeed, a number of providers in our sample were unfamiliar with government systems and some reported no familiarity with local offices and officials. However, while a number of providers suggested that the Kenyan government used to be hostile towards private providers, several also suggested that this attitude has been steadily changing in recent years.

*Respondent: I mean, before [government officials] didn’t use to offer us commodities. Today we are getting the commodities. They have moved that fear of when they come, they are coming to prosecute. They have removed that fear [and now] when they come, they are coming to work with me.*

                          (Nurse at an Amua clinic, Nairobi)

Due to an initial lack of familiarity with the government that made the NHIF accreditation process feel especially intimidating, providers were sometimes discouraged from even beginning the application process. Establishing a reputation with the government and in turn developing a relationship with local offices therefore proved an important first step for private providers by both creating and strengthening a previously tenuous connection.

### Building provider confidence

As suggested above, participating in the AHME quality improvement interventions – social franchising and SafeCare – helped providers feel more prepared to apply for NHIF accreditation and made the process feel less intimidating. Making improvements through SafeCare could translate to an easier accreditation experience. As one midwife at a Tunza clinic in Nairobi noted, *So, you find that what NHIF required, we had already implemented through SafeCare.*

By giving private providers the tools to ensure quality well before they sought out NHIF accreditation, the AHME partners helped these providers feel prepared for the application process, which in turn made the process less intimidating overall. In addition, providers felt they were able to achieve accreditation more easily after having participated in the interventions, because they had already anticipated and addressed potential roadblocks. These interventions therefore tempered the effects of street-level bureaucracy by empowering providers both to feel as though they could handle the system and to prepare their facilities well enough that there was less room for bureaucrats to wield their own power unnecessarily. 

### Complexity and confusion

Transparency and consistency effect the behavior of both the regulators and the regulated, creating or reducing loopholes, opportunities for corruption, and guidance on what one “should” do. Lack of transparency and consistency become both a barrier to effectiveness and an opportunity for front-line government workers to take shortcuts through the institutional red tape as a means to better carry out the mandates of their jobs. According to the AHME representatives who engaged with NHIF officials on behalf of providers, street-level bureaucrats in this context are most challenged not by deciding which regulations to ignore in order to get things done (the common OECD challenge), but rather by keeping up with a shifting policy landscape, and trying to determine which regulation to follow and how. As one franchise representative said:

*The health market in Kenya has been very dynamic for the last six months, especially with regard to NHIF accreditation. At one point … there was only one license that was required, now they want several licenses. So, that consistency has made us any time we are visiting the provider we come up with new updates. There has not been that consistency… Yes, the changes have been positive, but at the same time very aggressive, because you make one step and you are told you need another thing.*

                          (Franchise representative)

To keep up with the flow of new and varied regulations, representatives initiated bi-weekly meetings and an active WhatsApp group where both representatives and government agents regularly shared news. As another representative described it:

*The policy environment is changing very fast at the NHIF level and the government level. AHME has been changing with policy environment, so we keep on changing depending on the policy environment every year. And right now there are new changes with regard to the accreditation and that has been challenging because you need to keep pace and make sure that you are up to the changing environment. It’s not static.*

                          (Franchise Representative)

For the providers, a constantly changing environment and uncertainty around which rules applied at which point caused anxiety and made many of them hesitate to apply for NHIF accreditation in the first place. Further, those who had applied complained that NHIF officials did not follow up with feedback after conducting a clinic assessment; a step that would have helped the providers make required improvements and become accredited more quickly. NHIF officials also reportedly lost paperwork, forcing providers to submit multiple applications, and applied rules unevenly. This inconsistency and lack of transparency in the accreditation process created a space where providers may *prefer* paying bribes or feel this is their only option because it implies some certainty in an uncertain context.

*So, the experience [of applying for accreditation] was not good in that... they don’t follow the qualifications [criteria for selection]. That is what I can say. But if you go to their office and bribe them - there were others who just went to their offices [and bribed them] and in less than three months or four months - I was even told by a guy from [an informal settlement] that the officers who came were given the money.*

                          (Dentist (in-charge) at a Tunza Clinic, Nairobi)

### Cutting red tape

In addition to the practical solutions the AHME partners offered providers through the NHIF accreditation assistance program, some providers appreciated simply having a hand to hold through the accreditation process and noted that they were able to “walk together” with the franchise. This handholding worked.

***Interviewer: Has Tunza assisted [with NHIF accreditation] in any way?*** *Respondent: Yeah, they have…Uummh, the lady who was here was very, in fact she really pushed me very far. She even took the forms. She was taking them….as I fill the forms she could take them to NHIF and do the follow up until she made sure they have come…She did put a lot of effort, yes.*

                          (Nurse at a Tunza clinic, Central)

The providers who received accreditation assistance from the AHME partners appreciated that the partners liaised with the government on their behalf and gave them a hand to hold through a process that felt confusing and overwhelming. By acting as an intermediary in this way, the partners helped to demystify government processes for the private providers while also smoothing a path to accreditation that was more transparent and consistent, and less riddled with corruption.

## Discussion

Since private providers offer such a large proportion of outpatient services in many LMICs ( Grépin, 2016) ( Chakraborty & Sprockett, 2018), increased engagement between governments and private providers is a requisite step as countries around the world create or expand payment of private practitioners as a way to advance toward UHC. In Kenya, this engagement is growing at the same time that devolution within the health system is still in transition and is creating county-level governance systems that are in flux ( Tsofa *et al.*, 2017). Combined with a health policy landscape that also constantly shifts, new layers of bureaucracy created through devolution generate conditions in which rules and regulations that may be in conflict with each other constantly have to be parsed and interpreted by low-level bureaucrats who work directly with providers. While these conditions may create new opportunities for corruption ( D’Arcy & Cornell, 2016), and rent-seeking does occur, other motivations beyond greed matter more as both health providers and government officials need to navigate a complex and constantly shifting regulatory system on a day-by-day basis. 

Since this study was qualitative, our findings are not meant to be generalizable. However, our results show that an intermediary agency can help providers to navigate the regulatory landscape more quickly and successfully. A number of aspects of the AHME initiative have been called out by providers as especially helpful. These include encouragement to enter into NHIF accreditation processes even when those processes were not transparent, and support given to providers that helped to create positive relationships with the administrators responsible for interpreting and enforcing the new NHIF regulations. Providers also appreciated the concrete preparations for quality assessment by NHIF, which came with the AHME-supported SafeCare accreditation processes providers had already undertaken. At a time of regulatory transition as providers first enroll with NHIF, first pass their accreditation assessments, and increase the depth of their engagement with government, the intermediary services provided by the AHME network of organizations appears to have facilitated changes which would have been otherwise more difficult or perhaps not happened at all.

### Study limitations

The major limitation of this study is that the perspective of street-level bureaucrats themselves (e.g. frontline NHIF accreditation officers) is not represented. Since the data for this study is derived from an evaluation of the AHME program and these officials were not directly involved in AHME, they were not included in data collection. However, we have included the perspectives of franchise representatives who worked directly with these low-level NHIF officials in an attempt to represent some of the structural challenges these bureaucrats faced in their daily work. Our findings are not representative of every change in the government-private provider relationship that has taken place in Kenya during the past half-decade, nor can the changes be attributed to the work of AHME partners with certainty. Kenya has 47 counties and the changes beyond our sites, and in rural areas in particular, may have behaved quite differently from what we found. Our interviews took place over four years, but Kenyan financing and regulatory systems have continued to evolve, and what we have described here can only provide a description of what happened in a single period. Lastly, the data presented from providers below also may be affected by courtesy bias. Since almost all of the providers were part of an AHME-supported franchise network and understood the interviewers to be affiliated with AHME, they may have felt pressured to respond positively when prompted for their experience with the program. However, given that some of the AHME interventions, such as SafeCare and the expanded franchise support for NHIF accreditation, were offered to providers for free, it is unsurprising that providers would view such a package positively. 

## Conclusions

Our research shows the value of intermediary organizations in smoothing the path towards effective engagement between private providers and a national social health insurance system in Kenya. The partnership of NGOs we studied served to reduce confusion and misunderstandings, and to facilitate and encourage participation in national programs by private providers. All while also smoothing the bumps inherent in engaging with low-level bureaucracy as new policies are introduced and new systems for enrollment, accreditation, payment, and quality auditing are all rolled out.

In OECD health systems intermediaries are so common that they often go un-noted: the American, British, and German Medical Associations set standards and test for medical licensure and this licensure is in turn accepted by governments for approval of practice and reimbursement by national insurance. Hospitals are accredited by the Joint Commission in the US, the Japan Council for Quality Health Care in Japan, and Accreditation Canada in Canada. Again, governments in each country rely on these accreditation institutes and use their approval as a basis to allow facilities to operate or to be reimbursed by government funds. Similar intermediary agencies are active in lobbying for, representing, or supporting the engagement of dentists, ophthalmologists, podiatrists and other specialists; pharmacists, blood banks, and medical equipment suppliers; and nearly every other set of agencies across the vast panoply of actors in the health field.

Health systems are complex and the many actors who make up a system are themselves adaptive to changes, as our work has shown. Intermediaries are useful and facilitate changes while reducing transactional and rent-seeking costs. We observed the benefits of intermediaries in the Kenyan context to come primarily through increasing accountability and improving and speeding up communication between sectors of the health system. This is a particularly important role as new rules are created and implemented, and then clarified and adjusted iteratively while becoming common practice. Earlier in this paper we made a distinction between motivations of personal profit (corruption) and the other motivations of street-level bureaucrats which center around being effective in their work. The role of intermediaries that we see as being most important in Kenya addresses this second motivation of street-level bureaucrats: making their work easier and increasing their effectiveness.

Whether intermediaries are needed for the long-term functioning of a health system is a question which was not addressed in our work. However, we imagine that any intermediary roles which do continue would serve a different need over time as systems work more efficiently and both government and private actors understand the rules and each other better. Street-level bureaucracy is inherent in any governmental system, but the need to address it becomes less urgent as the systems become known and behaviors within them become normalized.

## Data availability

### Underlying data

The study Consent forms preclude sharing of interview transcripts beyond immediate research members. Attempts to revise these to allow de-identified transcripts from later survey rounds to be shared were not permitted by the Kenyan Institutional Review Board. The Review Board of the University of California, San Francisco determined that the wording of the Consent form from 2011 prohibits transcripts and data within analysis software from being shared outside of the research team. Our Consent Form is provided as extended data and relevant excerpts from the data are included in the body of the manuscript.

### Extended data

DRYAD: Qualitative survey instruments for a study on equity from a large-scale private-sector healthcare intervention in Ghana and Kenya: the African Health Markets for Equity (AHME) study. https://doi.org/10.7272/Q6FX77NG ( Montagu & Suchman, 2020).

This project contains the following extended data:

- Consent_Form_Kenya_Verbal.pdf ( *IRB approved form for Verbal Consent in Kenya)*
- Consent_Form_Written_and_Verbal.pdf ( *IRB approved form for Consent, both Kenya and Ghana. Includes option for verbal consent)*
- Guide_FGD_Community_Member_Female_2013.docx ( *Guide for women-only focus group survey)*
- Guide_FGD_Community_Member_Male_2013.docx ( *Guide for men-only focus group survey)*
- Guide_IDI_Franchise_Patient_2013.docx ( *2013 patient interview guide)*
- Guide_IDI_Franchise_Patient_2016.docx ( *2016 patient interview guide)*
- Guide_IDI_Franchise_Patient_2017.docx ( *2017 patient interview guide)*
- Guide_IDI_Franchise_Patient_Ghana_2018.docx ( *2018 patient interview guide, Ghana)*
- Guide_IDI_Franchise_Provider_2013.docx ( *2013 provider interview guide)*
- Guide_IDI_Franchise_Provider_Kenya_2017.docx ( *2017 provider interview guide, Kenya)*
- Guide_IDI_Franchise_Provider_Kenya_2018.docx ( *2018 provider interview guide, Kenya)*
- Guide_IDI_Franchise_Provider__Ghana_2018.docx ( *2018 provider interview guide, Ghana)*
- Guide_IDI_Implementer_2019.docx ( *2019 implementer interview guide)*
- Guide_IDI_NHI_2019.docx ( *2019 interview guide, Nat. Health Insurance staff)*
- Guide_IDI_Non-Franchise_Provider_Kenya_2017.docx ( *2017 provider interview guide, non-Franchise, Kenya)*
- Guide_IDI_Non-Franchise_Provider_Kenya_2018.docx ( *2018 provider interview guide, non-Franchise, Kenya)*
- Guide_IDI_Stakeholders_2013.docx ( *2013 stakeholder interview guide)*
- Guide_IDI_Stakeholders_global_2019.docx ( *2019 international stakeholder interview guide)*

Data are available under the terms of the Creative Commons Zero "No rights reserved" data waiver (CC0 1.0 Public domain dedication).
