# Recognizing and mediating bureaucratic barriers: increasing access to care through small and medium-sized private providers in Kenya

**DOI:** 10.12688/gatesopenres.13313.2

**Published:** 2021-10-15

**Authors:** Lauren Suchman, Edward Owino, Dominic Montagu

**Affiliations:** 1 University of California San Francisco, San Francisco, CA, USA; 2 Independent Consultant, Nairobi, Kenya

**Keywords:** Health financing, health insurance, evaluation, policy implementation, private providers, Kenya

## Abstract

**Background: **Equitable access to health services can be constrained in countries where private practitioners make up a large portion of primary care providers. Expanding purchasing arrangements has helped many countries integrate private providers into government-supported payment schemes, reducing financial barriers to care. However, private providers often must go through an onerous accreditation process to enroll in these schemes. The difficulties of this process are exacerbated where health policy is changed often and low-level bureaucrats must navigate these shifts at their own discretion. This paper analyzes one initiative to increase private provider accreditation with social health insurance (SHI) in Kenya by creating an intermediary between providers and “street-level” SHI bureaucrats.

**Methods:** This paper draws on 126 semi-structured interviews about SHI accreditation experience with private providers who were members of a franchise network in Kenya. It also draws on four focus group discussions conducted with franchise representatives who provided accreditation support to the providers and served as liaisons between the franchised providers and local SHI offices. There was a total of 20 participants across all four focus groups.

**Results:** In a governance environment where regulations are weak and impermanent, street-level bureaucrats often created an accreditation process that was inconsistent and opaque. Support from the implementing organizations increased communication between SHI officials and providers, which clarified rules and increased providers’ confidence in the system. The intermediaries also reduced bureaucrats’ ability to apply regulations at will and helped to standardize the accreditation process for both providers and bureaucrats.

**Conclusions:** We conclude that intermediary organizations can mitigate institutional weaknesses and facilitate process efficiency. However, intermediaries only have a temporary role to play where there is potential to: 1) directly increase private providers’ power in a complex regulatory system; 2) reform the system itself to be more responsive to the limitations of on-the-ground implementation.

## Introduction

The United Nations Sustainable Development Goals (SDG) call for all countries to achieve Universal Health Coverage (UHC) by 2030. Achieving this goal will require expanding both health financing and service availability. Many low- and middle-income countries (LMICs) are depending on expanded national budget allocations, and new social health insurance (SHI) schemes to better align health financing with UHC priorities. However, the extent to which these schemes can effectively advance progress toward UHC will depend on governments’ ability to contract with a sufficient number of health care providers and create a pool of quality service delivery options that are geographically and financially accessible. In sub-Saharan Africa, where private facilities provide almost half of the outpatient health services offered ( Chakraborty & Sprockett, 2018; Grépin, 2016), ensuring that private providers are accredited with local SHI systems is vital to achieving UHC. However, with SHI schemes facing such challenges as low re-enrollment rates ( Agyepong *et al.*, 2016) and a lack of transparency from government ( Abuya *et al.*, 2015) there may be few incentives for private providers to join. One of the key barriers to private provider participation in SHI schemes relates to the accreditation process itself; while public facilities are automatically enrolled into SHI schemes, private providers must go through a cumbersome formal accreditation process that often discourages providers from applying at all ( Sieverding *et al.*, 2018). Identifying the key roadblocks private providers face in the accreditation process and finding ways to ease the burdens of bureaucratic functions is likely to be a key determinant of expanding affordable and accessible healthcare services in support of UHC.

Drawing on interviews with small and medium-sized private providers, and NGO representatives in Kenya, an LMIC country that has recently expanded SHI contracting, this paper analyzes a programmatic effort to increase small and medium-sized private provider accreditation by mediating between providers and SHI officials. According to Lipsky, high-level policies often are re-worked on the ground where low-level bureaucrats interact with the public, reinterpreting broad or vague policies to address the immediacy of changing daily circumstances ( Lipsky, 1980). Our study looks at an intervention to reduce the variations in these “street-level” applications of policy by assisting private providers to enroll with the SHI scheme and, as a result, increase the number of healthcare service delivery points available to insured populations.

### Literature review

Private providers in LMICs often have minimal and conflicted relationships with the government systems that license, regulate, and sometimes contract them. Indeed, regulations specific to private practice in these settings are frequently limited and enforcement systems are weak ( Batley, 2006). Where such systems are effective at reaching private providers on a regular basis, providers rarely see these systems as beneficial, but rather as an imposition, often to be circumscribed or avoided altogether ( Montagu & Goodman, 2016).

***Regulatory complexity.*** Making the relationship between private providers and government all the more fraught, the complexity of regulations governing the private health sector in many LMIC settings limits those providers who do want to comply with the law and constrains their ability to do so. For example, an unintended effect of the widespread devolution of health system regulations in the early 2000s was the creation of more layers of bureaucracy, each empowered to develop its own laws and enforcement systems ( Cobos Muñoz *et al.*, 2017; Saltman & Bankauskaite, 2006). In some cases, this increased the regulatory burden on private providers, while in worst-case scenarios new regulations contradicted existing regulations. As a result, providers were inevitably doing something forbidden no matter which rule they elected to follow.

One result of this kind of regulatory complexity is that all actors in a system are eventually operating outside of the rules of one layer of government or another and are therefore susceptible to: incurring a host of new operating costs to become compliant; engaging in administrative or legal efforts to clarify guidance; or making unauthorized payments in order to avoid enforcement of rules ( Kisunko *et al.*, 1999). As regulations proliferate, those who enforce the rules, such as low-level bureaucrats, become increasingly powerful and autonomous ( Ramiro *et al.*, 2001). Where multiple unclear and contradictory rules exist, any enforcement decision by a local administrator or program officer can often be justified and the incentives to not participate grow, reducing both formal, and overall, care quality and availability.

Regulatory complexity can open the door for both government officials and citizens trying to navigate regulatory systems to exploit situations where rules are conflicting or unclear. As noted above, a common theme in LMICs is that policies exist, often many of them, but the legislation and regulatory guidance that clarify how policies are to be implemented and enforced are lacking ( Kaufmann, 1997). As Klitgaard has noted, unauthorized or hidden payments flourish in the absence of accountability, and complexity makes accountability more difficult ( Klitgaard, 1988). Indeed, studies have shown higher rates of unauthorized payments in countries with more tiers of government ( Fan *et al.*, 2009). Further, research has shown that the large information asymmetry between providers and patients, and the complexities that arise when payers’ incentives diverge from both providers and clients, all conspire to make healthcare systems particularly susceptible to payment irregularities and inefficiencies ( Mostert *et al.*, 2015; Vian, 2008). It is notoriously difficult to determine what counts as unauthorized or irregular in a complex system where opportunities to request payments can exist from the systemic and institutional levels down to the level of the individual, making it notoriously difficult to define ( Johnston, 1996). However, while much literature focuses on unauthorized payments requested from the government side ( Rose–Ackerman, 2008; Wang *et al.*, 2020; Zhang *et al.*, 2019), we note that such payments often are a two-way street in the context of regulatory complexity. Not only might low-level bureaucrats who have regular direct contact with the public take the opportunity to request bribes in order to make certain kinds of work worth their time, but citizens themselves may proactively offer bribes if they have reason to believe the system will be too difficult to navigate otherwise ( Miller, 2006).

***Street-level bureaucracy.*** In the context of complex and confusing regulatory systems, Lipsky’s concept of “street-level bureaucracy” is particularly relevant. According to Lipsky’s theory, low-level bureaucrats who work directly with the public re-work policy on the ground when they face situations where their institutions may not offer the support, resources or consistency they need to do their jobs well ( Lipsky, 1980). As Brodkin notes, street-level bureaucracy theory must be understood in the context of several key propositions that align closely with the context of the study analyzed in this paper. Underlying the theory of street-level bureaucracy, first, is the idea that policy is process rather than a fixed entity; this is most relevant when, as described above, policy is ambiguous and internally conflicted. In such cases, the actions of street-levels bureaucrats become policy in practice. However, these actions are not random, but are constrained and organized by bureaucrats’ working conditions. Bureaucrats’ responses to these conditions result in the systematic development of informal behaviors, which are particularly important because they shape not only policy, but also the relationship between the individual and the state ( Brodkin, 2012). These properties inherent to street-level bureaucracy can have both positive and negative consequences. On the one hand, some studies point to the importance of flexibility and a minimum level of decision-making power for low-level bureaucrats, which allows them to do their jobs more effectively ( Crook & Ayee, 2006) and compensate for the shortcomings of the health system writ large ( George, 2008). However, this flexibility also leaves room for cases in which bureaucrats do not understand a rule or how to apply it and, as suggested above, are left to interpret vague definitions at their own discretion ( Agyepong *et al.*, 2016). This may impede program implementation ( Kamuzora & Gilson, 2007), allowing street-level bureaucrats to, for example, implement a new policy according to their own values and views or to maintain a certain reputation in their community ( Kaler & Watkins, 2001; Walker & Gilson, 2004).

While requests for unauthorized payments commonly are cited as a significant barrier to provider participation in formal LMIC health systems, as outlined above, we suggest that the environment created by complex regulatory systems may be equally liable. Since the street-level bureaucrats who form policy in practice in these settings are almost unable to play by the rules, a lack of consistency and transparency in regulations and regulatory processes may hinder these processes just as much, if not more than, overt requests for bribes.

### The Kenyan context

While public sector providers in Kenya both have their salaries paid by government and work in government-financed facilities, private sector providers have limited interaction with government systems. These providers are expected to be licensed with the Kenya Medical Practitioners and Dentists Council (KMPDC) and to renew this license annually. However, licensing and regulation for the private health sector is fragmented and under-resourced, with one World Bank report referring to the regulatory arena as a “free for all” ( Barnes *et al.*, 2010). Private providers historically have had inconsistent interaction with the government at best and at worst hardly any interaction at all. However, this trend is changing as the country’s social health insurance scheme, National Hospital Insurance Fund (NHIF), continues to expand and opportunities for private providers to come into contact with government regulatory systems increase.

As in other countries where devolution has shifted governmental power dynamics, local administrators are now taxed with supervising licensure and regulatory issues as well as accreditation and payment through the expanded NHIF, giving them significantly more responsibility and authority than they had under the prior centralized system ( McCollum *et al.*, 2018; Obosi, 2019; Suchman, 2018). The motivation for devolution in Kenya has been to reduce corruption and increase responsiveness by bringing government closer to the people. However, constant shifts in policy are now filtered through several new levels of government before reaching providers on the ground. This creates a confusing and unpredictable environment for private primary health clinics.

In 2013, an NGO-led initiative began working to break through this tangle of new bureaucratic complexity.

### The African Health Markets for Equity (AHME) NHIF accreditation assistance intervention

The African Health Markets for Equity (AHME) initiative aimed to increase access to quality, private health care for the poorest populations in Kenya and Ghana. In Kenya, the program ran from 2012–2019 and incorporated social franchising through Marie Stopes Kenya (MSK) and Population Services Kenya (PS Kenya), and external quality accreditation systems through the PharmAccess Foundation. It also was designed to facilitate NHIF funding for poor populations being delivered through private primary care clinics. When AHME started, NHIF contracting in Kenya had only recently expanded to private clinics. Thus, most Kenyan providers were unaccredited and ineligible for reimbursement. In response to the low accreditation numbers, the social franchising partners developed an intervention that involved preparing the AHME-supported providers for NHIF accreditation inspection (including preparing paperwork, obtaining necessary licenses and conducting mock inspections), scheduling the inspection, and following up directly with NHIF officials to ensure that applications were vetted in a timely manner and feedback given to providers when necessary to fill gaps in their application. In addition, the franchisors set up quarterly forums between networked providers and NHIF officials, as well as a social media platform so that providers could connect directly with the officials. These forums were meant to build accountability, commitment and transparency from the NHIF side.

The findings detailed below were developed from the qualitative component of a mixed-methods evaluation of the AHME program. The objectives of this study were to document and analyze participating providers’ experiences with the AHME package of interventions, including the NHIF accreditation assistance intervention.

## Methods

Data for this paper were collected as part of the qualitative evaluation of the African Health Markets for Equity (AHME) program, which was conducted by the University of California San Francisco (UCSF). The AHME intervention package included social franchising enrollment, ( Viswanathan *et al.*, 2016) a quality improvement/quality accreditation initiative (see www.safe-care.org for more information), and access to loans for facility improvement or expansion (see www.medicalcreditfund.org/ for more information). In order to make these quality services more affordable for low-income populations, the AHME partners (Marie Stopes International and Marie Stopes Kenya, Population Services International and Population Services International Kenya, the PharmAccess Foundation, and formerly the International Finance Corporation) worked with the NHIF to identify people living in poverty and enroll them into the NHI scheme for free. The partners then applied the intervention described above to ease the accreditation process for providers so that they could serve the low-income patients now covered by NHIF at an affordable cost. Participating providers included all providers in the AHME-supported franchise networks who wished to pursue NHIF accreditation, but were not yet accredited.

### Sampling

***Providers.*** This analysis draws from a dataset of 126 semi-structured interviews with private providers in Kenya. This includes 24 interviews conducted in 2013, 52 interviews conducted in 2015 and 50 interviews conducted in 2017. This sample size was determined according to the sample size selected for the quantitative component of the mixed-methods evaluation and shifted over time as more clinics were enrolled into the AHME franchising intervention, ultimately representing approximately 50% of all franchised clinics. Since we required that participating providers had to have joined the franchise during the AHME intervention period, the sampling universe shifted somewhat from year to year. For the most recent round of data collection conducted for this study, MSK provided a list of 71 and PS Kenya provided a list of 45 recently franchise facilities. In addition, both franchises provided short lists of providers who had recently been approached to join the franchise, but had declined. From these lists we selected 15 MSK facilities, 15 PS Kenya facilities, and 20 non-franchised facilities to participate in the study aiming for a distribution of providers across geography and range of experience with the AHME interventions. For the purposes of this study, individual health facilities, not physicians, were considered “providers” and although there was minimal overlap in sampling across rounds of data collection, two facilities were visited more than once (both Rounds 2 and 3). However, because identifying information was not collected for interview participants, we have no way of confirming if the same person from these facilities participated in more than one interview. The qualitative dataset consists of semi-structured interviews with nurses, midwives, doctors, clinical officers, and other key decision-makers at private health facilities that were members of one of the AHME partner social franchises, as well as facilities that had been approached to join the franchise network but declined. In most cases, only one person was interviewed at each facility.

During each round of data collection, the AHME social franchising partners, MSK and PS Kenya, provided the research team with lists of providers franchised under the Amua (MSK) and Tunza (PS Kenya) networks. During Rounds Two (2015) and Three (2017) of data collection, the franchise partners also provided lists of providers who had been contacted to join the franchise, but had declined. These clinics were included in the sample to provide a point of comparison against which the research team could better determine the effects of the AHME interventions.

Using the provider lists provided by MSK and PS Kenya, we used a purposeful criterion sampling strategy ( Palinkas *et al.*, 2015) to design a sample that represented providers with a mix of experiences with the AHME intervention package. In order to capture potential effects of the NHIF accreditation assistance intervention, we also selected facilities based on their NHIF accreditation status in Rounds 2 and 3 (2015 and 2017). Interviews were conducted with providers in a range of facility types across six regions (Nairobi, Eastern, Coast, Central, Rift Valley, Kajiado) during the three rounds of data collection.

All potential participants in a franchise network were made aware of the study by the program implementers (the franchising organizations) and then approached in person by a member of the research team who invited them to join the study. Almost all franchised providers agreed to be interviewed after being approached. The non-franchised providers were approached directly by the research team and invited to participate in an interview. Refusal rates for this population were not available at the time this paper was written. In order to reduce potential bias in the sample we attempted to make it clear that the research team was independent from the program implementing partners when approaching providers. In addition, field staff were trained in qualitative interviewing techniques specifically meant to reduce bias, such as asking open-ended questions and responding to interviewees with neutral expressions.

***Franchise representatives.*** In addition to interviews with private providers, this analysis draws from focus group discussions (FGD) conducted in 2018 with franchise representatives who worked with the AHME-supported providers. These representatives were staff at either MSK or PS Kenya and acted as liaisons between the providers and NHIF officials, helping providers to prepare for accreditation and then working with the NHIF officials to ensure that these applications moved along quickly and smoothly. Focus groups with franchise representatives were conducted only in 2018 in order to provide context for the AHME qualitative evaluation team as they concluded their analysis. To select FGD participants, the AHME implementing organizations were contacted and asked to provide the names of at least three franchise representatives who would be willing to talk with the qualitative evaluation team with the aim of conducting two FGDs at each organization each with at least three participants. A total of four focus group discussions were conducted (two at each organization) with a total of 20 participants across all four groups. All of the potential participants who were approached agreed to participate.

### Tools

*Providers*. During each round of data collection, interviewers used a semi-structured interview guide that had been written by the research team at UCSF and piloted by IPA using franchised providers who were eligible for the study. Piloting in each round resulted in small changes to the wording of certain questions, but no substantive changes to the overall guide. Providers were asked about their experiences with the AHME interventions and their knowledge of or desire to join any interventions in which they were not currently participating. In Rounds Two and Three (2015, 2017) of data collection, providers also were asked about their perceptions of and experiences with the NHIF. All guides and consent forms can be found as extended data ( Montagu & Suchman, 2020).

*Franchise representatives.* Given that the population of franchise representatives was relatively small, the focus group discussion guide was not piloted. However, the discussion guide was intentionally left flexible so that discussion leaders could respond to topics and themes as they arose. Topics included the representatives’ daily responsibilities, the nature of their relationships with providers, and details of their work with the NHIF. All guides and consent forms can be found as extended data ( Montagu & Suchman, 2020).

### Data collection and processing

***Providers***. The UCSF team partnered with Innovations for Poverty Action (IPA), a research organization based in New Haven, CT with country offices across the globe to collect provider data in Kenya. IPA recruited field interviewers who were then trained by the UCSF team working with IPA staff.

Data collection with providers took approximately one month during each round. Field staff traveled to clinics where providers had already been contacted by IPA and agreed to participate in an interview. However, providers did not have additional information about the interviewer they would be working with ahead of time. Upon arriving at the interview site, interviewers confirmed that the interviewee was one of the key people at the facility who had been involved in decision-making regarding whether or not to participate in the AHME interventions. They then obtained informed consent from the providers prior to conducting semi-structured interviews that lasted approximately 60 minutes each. Interviews were most often conducted at the health facility where the provider worked. Where possible, interviews took place in a private consulting room or office at the health facility to ensure privacy and data quality.

All interviews were recorded using digital recorders in the language the interviewee was most comfortable using. In anticipation that all respondents would not be comfortable conducting a full discussion in English, interview guides were first developed in English and then professionally translated into Swahili to ensure that the translations accurately captured the intended meanings of the original guide. In addition, IPA field staff were all Kenyan and native Swahili speakers. Recordings were translated and transcribed simultaneously by a team of professional Kenyan transcriptionists who had been trained on key terms. IPA research assistants were responsible for back-checking interviews, including ensuring translation accuracy. After the back-checking process was concluded, IPA transferred the transcripts to UCSF for analysis. Transcripts were not returned to participants for comment.

***Franchise representatives.*** Data collection with franchise representatives took place in June 2018 and all of the focus group discussions were conducted by the UCSF team, which consisted of a PhD-level researcher and a program manager, both with experience conducting qualitative interviews and focus groups. Focus group participants were debriefed ahead of time by their supervisors who shared the purpose of the study. The FGDs were held in a private meeting room at either the MSK or PS Kenya offices. The UCSF team obtained verbal informed consent from all participants before starting discussion, each of which lasted approximately 90 minutes.

All FGDs were recorded using digital recorders and all were conducted in English, which was familiar to all participants. Recordings were transcribed by a professional Kenyan transcriptionist who had been trained on key terms and were back-checked by UCSF staff. Transcripts were not returned to participants for comment.

### Data analysis

All transcripts were coded by two researchers from the UCSF team with assistance from two IPA research assistants during the final round of data analysis with the exception of the FGDs, which were coded solely by one UCSF researcher due to the complexity of coding FGD transcripts. Coders used the popular qualitative analysis program Atlas.ti version 8. Open source alternatives to Atlas.ti include Qualcoder and RQDA. Dedoose is a paid, but lower-cost alternative. The UCSF team used an inductive, thematic approach to coding and analyzing the interviews. This was because there was little existing literature on private providers’ experiences with social health insurance in general and with the Kenyan NHIF specifically from which to derive prior theories.

An initial coding scheme was created in 2013 based on thematic coding of a sub-set of the interviews from each country and each interview was coded using an open coding approach, in which codes were derived from the data. Common codes were identified across the interviews and grouped into code families and sub-codes. Codes aligned with the main themes of the evaluation, specifically provider experiences with each of the AHME interventions, challenges and benefits of the interventions, and provider experiences with NHIF accreditation. During subsequent rounds of analysis, codes were refined to allow for new priorities in analysis while ensuring continuity across rounds. New codes were developed, also inductively, for the single round of franchise representative FGDs.

The coding team reviewed the codebook together during each round of analysis to ensure common understanding of codes and consistency in application. During each round of coding, coders jointly coded 2-3 transcripts and discussed questions and discrepancies to determine inter-coder reliability before beginning independent focused coding. The first author also reviewed a sub-set of coded interviews during each round to check for consistency across coders. The coding process indicated that saturation was reached for themes related to NHIF experience and both preliminary and final findings were shared with the participants and the implementing organizations. The implementing organizations had the opportunity to comment on the preliminary findings and the research team took these comments into account while preparing final documentation while taking care to maintain the integrity of the external evaluation.

### Ethical approvals

Ethical approval for the AHME qualitative evaluation was provided for each round of data collection by the Kenya Medical Research Institute (Protocol #Non SSC no. 411), and with “exempt” status from the Institutional Review Board of UCSF. According to the requirements of the KEMRI IRB, informed verbal consent was obtained from clients in Kenya before interviews were conducted. Providers were given the option to withdraw their participation at any time with no consequences for their participation in the AHME interventions. To thank them for their time, participating providers were given a small gift worth approximately five US dollars, such as a pack of rubber gloves.

## Results

As shown in Table 1, the providers interviewed for this study were largely in their 40s and had been in practice for approximately 20 years. While we interviewed more women in the first round of interviewing, subsequent rounds included an almost even split of women and men (2015) with significantly more men interviewed in 2017. Across all rounds of data collection, most of those interviewed were nurses with relatively few doctors and midwives included in the sample. Since AHME focused its efforts on smaller private providers most facilities visited were clinics, although these facilities could range in size from one room to several. Further, because data collection focused on the person at each facility who was responsible for key decision-making, the sample is largely made up of facility owners across all rounds. 

**Table 1.** Provider sample characteristics. NHIF=National Hospital Insurance Fund; AHME=African Health Markets for Equity.

	2013	2015	2017
**Number of** ** interviews**	24	52	50
**Average age**	49 years old	47 years old	41 years old
**Males**	9	31	32
**Females**	15	26	18
**Average years in** ** practice**	21 years	20 years	17 years
**Owners of facility**	75%	74%	68%
**Region**			
Central	0%	0%	18%
Coast	0%	0%	18%
Eastern	71%	32%	12%
Nairobi	29%	37%	16%
Nyanza	0%	0%	28%
Rift Valley	0%	31%	8%
**Provider title**			
Medical Doctor	8%	9%	8%
Nurse	42%	42%	30%
Community Health/ Auxiliary Nurse	17%	9%	7%
Midwife	4%	2%	11%
Clinical Officer	21%	17%	28%
Other/Unknown	8%	21%	16%
**Facility type**			
Clinic	71%	62%	50%
Hospital	17%	9%	16%
Health Center	8%	8%	10%
Maternity Home/ Nursing Home	4%	19%	6%
Pharmacy	0%	2%	8%
**AHME ** **interventions**			
Amua	42%	48%	30%
Tunza	58%	40%	30%
NHIF accreditation	37%	35%	38%
Quality improvement intervention	n/a	38%	56%
Loans and business support	n/a	21%	54%

Franchise representatives included in the study generally worked with providers in one of three roles: direct liaison regarding franchising and quality improvement, business support, and health financing liaison. Each type of representative described having a particular role to play in helping providers to understand the value of NHIF accreditation and ultimately completing the process. Business support officers discussed with the providers the opportunities available for financing quality improvement in their facilities and the potential return on investment, while franchise officers provided support for implementing quality improvement interventions, such as SafeCare. Health financing liaisons helped providers to usher their applications for accreditation through the system through direct communication with contacts at NHIF. 

While the street-level bureaucrats themselves were not included in our data collection, our sampling allows for a triangulation of opinions about their role from the private stakeholders affected by their work. These bureaucrats are generally in charge of assessing whether public or private facilities have met the minimum qualifications for accreditation, and also are responsible for ensuring that these facilities are compliant with the necessary statutory requirements. These officials sit within the devolved health units, making it easier for them to communicate new regulations and policies to the private providers given their geographic proximity.

### Building a relationship & reputation with government

Many providers in our sample said they were unfamiliar with government systems and some reported no familiarity with local offices and officials to begin with. However, while a number of providers suggested that the Kenyan government used to be hostile towards private providers, several also suggested that this attitude has been steadily changing in recent years.

*Respondent: I mean, before [government officials] didn’t use to offer us commodities. Today we are getting the commodities. They have moved that fear of when they come, they are coming to prosecute. They have removed that fear [and now] when they come, they are coming to work with me.*

                                                                                                                                                 (Nurse at an Amua clinic, Nairobi)

This initial lack of familiarity with government was one factor that made the NHIF accreditation process feel especially intimidating, and providers were sometimes discouraged from even beginning the application process as a result. However, some providers felt that joining a franchise network enabled them to establish a reputation with government, which was then strengthened through the relationships franchise representatives fostered with local NHIF offices. Not only did providers feel more confident engaging with government as a result, but as one franchise representative noted:

*I think for the first time for example especially for the provider, private provider, they feel like they are part of the health system, the Kenyan health system. Because for a very long time they [got only] some minimal support from the government at times.*

Providers corroborated this statement, noting that they had only been introduced to local government through their franchise representative. Indeed, as we have shown elsewhere ( Suchman *et al.*, 2018), NHIF officials may have been especially open to working with AHME-supported providers because the AHME partnership and the NHIF shared the same overall goal of increasing access to care for poor populations.

### Navigating regulatory complexity & systemic shortfalls

According to the AHME representatives who engaged with NHIF officials on behalf of providers, navigating a policy landscape that is both constantly shifting and internally inconsistent presents serious challenges for both providers and those who are seeing them through the accreditation process. Speaking about the rapidly changing health policy environment, one franchise representative said:

*The health market in Kenya has been very dynamic for the last six months, especially with regard to NHIF accreditation. At one point … there was only one license that was required, now they want several licenses. So, that consistency has made us any time we are visiting the provider we come up with new updates. There has not been that consistency… Yes, the changes have been positive, but at the same time very aggressive, because you make one step and you are told you need another thing.*

                                                                                                                                                 (Franchise representative)

As another franchise representative pointed out, not only is the policy environment constantly changing, but these changes are applied unevenly across local NHIF offices. While the representative noted that this inconsistency may be due to a lack of internal communication, they also posited that the local branches themselves might be changing policies as they come in. This results in a lack of consistency and clarity for the representatives themselves, who then have to communicate these policy changes to providers: 

*One of the other challenges we are having is that there are a lot of changes in NHIF. Today you wake up and they say these are the requirements, the following day its different. So, if you are working with like three counties each branch requires something different from the other, yet you are dealing with the same NHIF. So, that has been a challenge because as a [franchise representative] I am expecting that this is the processs, but then I go to another county and they tell me this is the process, I go to another one they tell you. So, there has been changes, yes, but it’s like they are not communicated all the way to all the branches or maybe they are communicated and they are changed. I am not sure.*

As noted in the intervention description above, AHME representatives initiated regular in person meetings and an active WhatsApp group where both private providers and NHIF official could follow up and exchange information about new regulations. Still, many providers were hesitant to engage in a complex process they didn't fully understand, often noting they were deterred by colleagues who had reported difficulties.

Among those who had applied, providers regularly reported encountering a system that felt opaque and inconsistent, and they sometimes suspected that NHIF officials were taking advantage of their ignorance to prolong the accreditation process. Indeed, many said the accreditation process took longer than expected and that they felt unsure about where their application was in the process at any given time or when to expect follow up. In fact, the NHIF does not have a specific system for communicating with providers throughout the accreditation process. This means that providers must rely on direct communication with NHIF officials if they want to track their own application. However, each official handles hundreds of accreditation cases and has limited capacity to respond to individual provider requests.

According to providers, NHIF officials also reportedly lost paperwork, forcing providers to submit multiple applications, and applied rules inconsistently. In addition to noting that NHIF officials are overwhelmed by their individual caseloads, which may help to explain the lost paperwork, one franchise representative who worked particularly closely with the NHIF also suggested that officials often were not trained or adequately prepared to carry out some of their most basic tasks, such as conducting clinic inspections. These systemic failures created an inconsistency and lack of transparency in the accreditation process at the same time that they made providers feel suspicious they were being exploited by the low-level bureaucrats. In this context, providers felt lost and sometimes were under the impression that it was necessary to pay a bribe in order to make the process work for them.

*So, the experience [of applying for accreditation] was not good in that... they don’t follow the qualifications [criteria for selection]. That is what I can say. But if you go to their office and bribe them - there were others who just went to their offices [and bribed them] and in less than three months or four months - I was even told by a guy from [an informal settlement] that the officers who came were given the money.*

                                                                                                                                                 (Dentist (in-charge) at a Tunza Clinic, Nairobi)

As illustrated in the above quote, it is worth noting that relatively few providers in our sample reported having paid bribes themselves, although several mentioned hearing from colleagues that they had paid bribes in order to become accredited. Combined with our findings on regulatory complexity and other challenges providers faced during the accreditation process, provider reports of bribery are particularly telling. We conclude that the inconsistent and opaque actions of low-level NHIF officials in this context create anxiety around dealing with the officials themselves, as evidenced by providers’ tendencies to believe colleagues who said they had paid bribes. This anxiety, coupled with a general lack of understanding of the process itself, can become a barrier to entry into the NHIF for small private providers.

### Cutting red tape

Participating in the AHME interventions helped providers feel more prepared to go through the accreditation process and also gave them a “hand to hold,” which both made the process feel less intimidating and practically eased the path to accreditation. By joining a social franchise, providers already received quality improvement support and, indeed, providers had to meet a minimum level of quality in order to remain franchised. Further, because the SafeCare guidelines are largely aligned with the NHIF’s accreditation requirements, providers who participated in SafeCare often found that they had an easier accreditation experience as a result. As one midwife at a Tunza clinic in Nairobi noted, *So, you find that what NHIF required, we had already implemented through SafeCare.*

By giving private providers the tools to ensure quality well before they sought out NHIF accreditation, the AHME partners helped these providers feel prepared for the application process, which in turn made the process less intimidating overall. In addition, providers felt they were able to achieve accreditation more easily after having participated in the interventions, because they had already anticipated and addressed potential roadblocks.

In addition to following concrete steps to improve quality and make accreditation easier, providers also appreciated having a partner to liaise with local NHIF offices and help them “push” their application through the bureaucracy. Further, the relationship between the franchise representatives and providers proved especially important for providers to feel that the accreditation process was manageable. It also helped providers feel someone was on their side in the face of a bureaucratic system that was otherwise out to impede their progress. Indeed, franchise representatives themselves regularly spoke of cultivating relationships with providers and enabling them to realize the full potential of their business:

*Nurturing them essentially is all about you will find a provider has a certain idea or this is how they do their things and maybe they are not – there is where they want to be. So in nurturing it’s all about sort of maybe you can do capacity building for them, you know like trying to make their idea grow. Like we help them grow. We help them live up to the dreams that they want. Live the ambitions that they have.*

                                                                                                                                                 (Franchise representative)

Both franchise representatives and providers also spoke of giving providers a “hand to hold” and “walking together” through an accreditation process that otherwise felt confusing and complex. While providers often felt overwhelmed by the amount of follow-up required to get an application approved, they recognized that partnering with a franchise representative helped to “push” the process forward more quickly than they could have managed on their own. 

*Interviewer: Has Tunza assisted [with NHIF accreditation] in any way?*

*Respondent: Yeah, they have…Uummh, the lady who was here was very, in fact she really pushed me very far. She even took the forms. She was taking them….as I fill the forms she could take them to NHIF and do the follow up until she made sure they have come…She did put a lot of effort, yes.*

                                                                                                                                                 (Nurse at a Tunza clinic, Central)

By acting as a link between providers and NHIF officials in this way, the partners helped smooth a path to accreditation that was more transparent and consistent than what the providers likely would have experienced otherwise. In addition, the franchise representatives eased the burden placed on NHIF officials managing large caseloads by streamlining processes and communications, and providing the technical assistance the officials themselves didn’t have the training or time to give.

## Discussion

Our findings suggest that the AHME interventions helped to ease the process of NHIF accreditation by simplifying and creating transparency in a process that otherwise felt overly complex and intimidating on the provider side, and also created a heavy burden for street-level NHIF bureaucrats. Through these interventions, the AHME partners helped to mitigate the effects of street-level bureaucracy by building connections between providers and NHIF officials, streamlining processes and communications on the NHIF side, and giving providers a familiar hand to hold through the accreditation process. However, while AHME was quite successful in facilitating NHIF accreditation for franchisees, as with many time-bound donor-funded projects ( Hofisi & Chizimba, 2013; Muluh *et al.*, 2019; Seppey *et al.*, 2017), it failed to create the transformative or sustainable change that could have resulted with more forethought and intention. While franchise representatives managed to forge strong alliances with the NHIF, and this was a major success of the project, these relationships were not institutionalized. This lack of institutionalization created a situation where relationships could easily fall apart when donor resources were no longer supporting them and project staff left participating partner organizations.

As we have noted elsewhere ( Suchman *et al.*, 2020), networks established by private providers themselves, rather than external organizations such as franchisors, have great potential to increase the visibility and power of small private providers. In theory, providers previously supported by AHME should be able to join private provider networks such as the Kenya Healthcare Federation (KHF), the Kenya Association of Private Hospitals, and the Rural and Urban Private Health Association (RUPHA) in order to maintain a relationship with the NHIF. However, most of these networks charge membership fees that are too high for small private providers to afford. The provider population that AHME originally set out to support therefore remains without an effective governance structure to help it interface with institutions such as the NHIF.

If implemented well, strong provider networks could help to reduce opportunities for street-level bureaucrats to take advantage of providers who are ignorant of the system and lack the relationships needed to track the accreditation process as it moves along. On the provider side, these networks could also offer members the support and accountability mechanisms they enjoyed through AHME, thereby helping them to cut the red tape of bureaucracy that makes the accreditation process feel so intimidating to many. 

In addition to recommending that established private provider networks in Kenya make accommodations to include smaller, less profitable facilities, we also offer several recommendations for the NHIF and donors in the health financing space to mitigate the effects of street-level bureaucracy. First, we recommend that the NHIF invest in updated trainings for officials who conduct site inspections to increase consistency in the accreditation process while also improving the quality of the facility’s services. Second, we suggest that the NHIF invest in an online platform that will allow providers to track the progress of their accreditation applications. This would both increase provider confidence and visibility into the system while also reducing the burden on low-level NHIF officials to communicate with all providers going through the accreditation process. Third, we recommend that donors in the health financing space invest in platforms and networks that increase communication between small private providers and the low-level bureaucrats who act as gatekeepers to participation in larger government structures. This would help providers to more effectively navigate a complex system themselves and, in turn, become more integrated into Kenya’s health system writ large. Finally, rather than placing the onus on individuals and individual institutions to fix the holes in a complex regulatory system, at a higher level we note that regulatory policy itself could be changed so that it is more responsive to on-the-ground implementation. Scholars working in India, for example, have recommended adopting a broader concept of regulation and enhancing the participation of key stakeholders in regulatory design to address similar issues of regulatory complexity ( Porter *et al.*, 2021).

### Study limitations

The major limitation of this study is that the perspective of street-level bureaucrats themselves (e.g. frontline NHIF accreditation officers) is not represented. Since the data for this study is derived from an evaluation of the AHME program and these officials were not directly involved in AHME, they were not included in data collection. However, we have included the perspectives of franchise representatives who worked directly with these low-level NHIF officials in an attempt to represent some of the structural challenges these bureaucrats faced in their daily work. Our findings are not representative of every change in the government-private provider relationship that has taken place in Kenya during the past half-decade, nor can the changes be attributed to the work of AHME partners with certainty. Kenya has 47 counties and the changes beyond our sites, and in rural areas in particular, may have behaved quite differently from what we found. Our interviews took place over four years, but Kenyan financing and regulatory systems have continued to evolve, and what we have described here can only provide a description of what happened in a single period. Lastly, the data presented from providers below also may be affected by courtesy bias. Since almost all of the providers were part of an AHME-supported franchise network and understood the interviewers to be affiliated with AHME, they may have felt pressured to respond positively when prompted for their experience with the program. However, given that some of the AHME interventions, such as SafeCare and the expanded franchise support for NHIF accreditation, were offered to providers for free, it is unsurprising that providers would view such a package positively.

## Conclusions

Our research shows the value of intermediary organizations in mitigating the effects of street-level bureaucracy by increasing meaningful communication and accountability between private providers and bureaucrats, and reducing the burden on both to navigate complex government processes. This is a particularly important role as new regulations are created and implemented, then iteratively clarified and adjusted while becoming common practice. Earlier in this paper we addressed the literature on unauthorized and hidden payments in conversation with the literature on street-level bureaucracy, and the ways in which bureaucrats may be forced to act outside of the regulatory system in order to get their job done. Since we had little reliable data on unauthorized payments, we cannot draw any firm conclusions about these practices. However, we believe that the role of intermediaries in Kenya best addresses a key motivation of street-level bureaucrats: making their work easier and increasing their effectiveness. Further, we note that an increase in communication, accountability and understanding between bureaucrats and providers is likely to reduce requests for unauthorized payments on both sides as well. 

Whether intermediaries are needed for the long-term functioning of a health system is a question which was not fully addressed in our work. However, we imagine that any intermediary roles which do continue would serve a different need over time as systems work more efficiently, providers gain more power, and both government and private actors better understand the rules and each other. Street-level bureaucracy is inherent to any governmental system, but the need to address it becomes less urgent as systems adapt to conditions on the ground, regulations become known, and behaviors on both the provider and bureaucrat sides become normalized.

## Data availability

### Underlying data

The study Consent forms preclude sharing of interview transcripts beyond immediate research members. Attempts to revise these to allow de-identified transcripts from later survey rounds to be shared were not permitted by the Kenyan Institutional Review Board. The Review Board of the University of California, San Francisco determined that the wording of the Consent form from 2011 prohibits transcripts and data within analysis software from being shared outside of the research team. Our Consent Form is provided as extended data and relevant excerpts from the data are included in the body of the manuscript.

### Extended data

DRYAD: Qualitative survey instruments for a study on equity from a large-scale private-sector healthcare intervention in Ghana and Kenya: the African Health Markets for Equity (AHME) study. https://doi.org/10.7272/Q6FX77NG ( Montagu & Suchman, 2020).

This project contains the following extended data:

- Consent_Form_Kenya_Verbal.pdf ( *IRB approved form for Verbal Consent in Kenya)*
- Consent_Form_Written_and_Verbal.pdf ( *IRB approved form for Consent, both Kenya and Ghana. Includes option for verbal consent)*
- Guide_FGD_Community_Member_Female_2013.docx ( *Guide for women-only focus group discussion)*
- Guide_FGD_Community_Member_Male_2013.docx ( *Guide for men-only focus group discussion)*
- Guide_IDI_Franchise_Patient_2013.docx ( *2013 patient interview guide)*
- Guide_IDI_Franchise_Patient_2016.docx ( *2016 patient interview guide)*
- Guide_IDI_Franchise_Patient_2017.docx ( *2017 patient interview guide)*
- Guide_IDI_Franchise_Patient_Ghana_2018.docx ( *2018 patient interview guide, Ghana)*
- Guide_IDI_Franchise_Provider_2013.docx ( *2013 provider interview guide)*
- Guide_IDI_Franchise_Provider_Kenya_2017.docx ( *2017 provider interview guide, Kenya)*
- Guide_IDI_Franchise_Provider_Kenya_2018.docx ( *2018 provider interview guide, Kenya)*
- Guide_IDI_Franchise_Provider__Ghana_2018.docx ( *2018 provider interview guide, Ghana)*
- Guide_IDI_Implementer_2019.docx ( *2019 implementer interview guide)*
- Guide_IDI_NHI_2019.docx ( *2019 interview guide, Nat. Health Insurance staff)*
- Guide_IDI_Non-Franchise_Provider_Kenya_2017.docx ( *2017 provider interview guide, non-Franchise, Kenya)*
- Guide_IDI_Non-Franchise_Provider_Kenya_2018.docx ( *2018 provider interview guide, non-Franchise, Kenya)*
- Guide_IDI_Stakeholders_2013.docx ( *2013 stakeholder interview guide)*
- Guide_IDI_Stakeholders_global_2019.docx ( *2019 international stakeholder interview guide)*
- Guide_FGD_Franchise Representatives_2018.docx *(Guide for franchise representative focus group discussion)*

Data are available under the terms of the Creative Commons Zero "No rights reserved" data waiver (CC0 1.0 Public domain dedication).
