# Supplementary material for: Recognizing and mediating bureaucratic barriers: increasing access to care through small and medium-sized private providers in Kenya
Source: Gates Open Res. 2021 Dec 6;5:95. Originally published 2021 Jun 17. [Version 3] doi: 10.12688/gatesopenres.13313.3 (PMC8649627; doi:10.12688/gatesopenres.13313.3)
Supplement: Peer review history

# Peer review: Reviewer response for version 3

**DOI:** 10.21956/gatesopenres.14740.r31520

**Published:** 2021-12-06

**Authors:** Meenakshi Gautham

**Affiliations:** 1 Department of Global Health and Development, Faculty of Public Health and Policy, London School of Hygiene & Tropical Medicine, London, UK

Approved from my end.

Is the work clearly and accurately presented and does it cite the current literature?

Partly

If applicable, is the statistical analysis and its interpretation appropriate?

Not applicable

Are all the source data underlying the results available to ensure full reproducibility?

No

Is the study design appropriate and is the work technically sound?

No

Are the conclusions drawn adequately supported by the results?

No

Are sufficient details of methods and analysis provided to allow replication by others?

Partly

Reviewer Expertise:

NA

I confirm that I have read this submission and believe that I have an appropriate level of expertise to confirm that it is of an acceptable scientific standard.

# Peer review: Reviewer response for version 2

**DOI:** 10.21956/gatesopenres.14665.r31242

**Published:** 2021-11-23

**Authors:** Meenakshi Gautham

**Affiliations:** 1 Department of Global Health and Development, Faculty of Public Health and Policy, London School of Hygiene & Tropical Medicine, London, UK

I'd like to thank the authors for addressing my comments. I enjoyed reading through the revised manuscript and am happy to recommend that it be accepted for indexing. I do still have a minor comment - there are a couple of quotes without the sources. Please could you add the sources for all quotes (and the source ID no.'s wherever it makes sense), just to demonstrate that you have used a variety of quotes. For example, ' *franchise representative 01, tunza clinics*'.

Is the work clearly and accurately presented and does it cite the current literature?

Partly

If applicable, is the statistical analysis and its interpretation appropriate?

Not applicable

Are all the source data underlying the results available to ensure full reproducibility?

No

Is the study design appropriate and is the work technically sound?

No

Are the conclusions drawn adequately supported by the results?

No

Are sufficient details of methods and analysis provided to allow replication by others?

Partly

Reviewer Expertise:

NA

I confirm that I have read this submission and believe that I have an appropriate level of expertise to confirm that it is of an acceptable scientific standard, however I have significant reservations, as outlined above.

# Peer review: Reviewer response for version 1

**DOI:** 10.21956/gatesopenres.14556.r30801

**Published:** 2021-07-23

**Authors:** Meenakshi Gautham

**Affiliations:** 1 Department of Global Health and Development, Faculty of Public Health and Policy, London School of Hygiene & Tropical Medicine, London, UK

**Overall comments:** This study addresses the critical issue of implementation gaps in the top-down regulatory architecture of many LMICs. The literature review is interesting and comprehensive but too discursive and it leaves little room for the findings. The analysis could have a lot more depth given that it is based on 126 semi-structure interviews and several FGDs. However I did not a convincing or cogent story emerging in the paper. The study aim is to show how the intermediary organisation addresses the barriers due to low level government workers’ issues but the findings have not fully engaged with or developed this theme. Partly this is because none of these street level bureaucrats (SLBs) have been interviewed, but even so, you could build a much more coherent story based on the responses of the recipient providers and the intermediaries. Tell us more about who these street bureaucrats are, how do they operate, what is the range of interactions that providers have with them, which interactions are the problematic ones, are there any issues that are commonly misinterpreted by the SLBs or are their actions random and arbitrary, when are bribes needed to be given, and what are the different tools/strategies through which the intermediaries/intervention deal with the different types of problems created by the laws and the SLBs. Is this a default outcome of the intervention or is it by design? Were there any differences in the way that the different provider types interacted with the SLBs? I feel that it would really help you to have a conceptual framework based on Lipsky’s theory, for your analysis to make greater sense of SLB behaviours and how the intervention helps providers deal with these behaviours.

 More detailed comments below:

**Results: **

- It would be very useful to begin with a description of the key actors involved in the scenario – the SLBs as well as the providers. You do tell us about the providers in the table - this could be summarised in the text drawing attention to the major provider types and the greater proportion of nurses and auxiliaries in the sample. Did you find any difference in the way that doctors coped with the regulations compared with nurses? If you found any differences, these could be unpacked and explained in the discussion.
- The findings are presented as very generalised statements rather than as qualitative evidence. For example: ‘Transparency and consistency effect the behavior of both the regulators and the regulated, creating or reducing loopholes, opportunities for corruption, and guidance on what one “should” do. Lack of transparency and consistency become both a barrier to effectiveness and an opportunity for front-line government workers to take shortcuts through the institutional red tape as a means to better carry out the mandates of their jobs. Can you present more direct evidence from the narratives that makes you arrive at this interpretation? Like (just a hypothetical example): ...several providers shared their experiences of having faced problems with SLBs like having their application forms rejected for accreditation for no apparent reason and not being available when approached for a clarification…(give a quote here...). This is especially important when you present evidence about corruption. It is not at all clear how the intervention has reduced or stopped this or made the path more ‘transparent and consistent’.

**Methods:**

- It would be very useful to have a short description of the context/setting and the interventions upfront as these are critical to the study/analysis and the reader should understand them clearly to make sense of the findings.
- What was the sampling universe? What was the total number of facilities of different types from where the sample was drawn?
- It would be useful to have all the information about the tools and the piloting brought together under a sub-section on tools.
- Please avoid long complex sentences like this one: "Upon arriving at the interview site, interviewers confirmed that the interviewee was one of the key people at the facility who had been involved in decision-making around whether or not to participate in the AHME interventions and obtained informed consent from the providers prior to conducting semi-structured interviews that lasted approximately 60 minutes each."

**Discussion:**

- I find that this section just repeats part of the introduction and the findings, without engaging with the findings. What would you say are your most important findings, what is their novelty, and how do you explain them? You need to go into greater depth to explain why intermediaries have been useful and I think this is why having a conceptual framework for this study would be very useful.
- I would also have questions about the implications of your findings. How sustainable is it to have intermediary organisations help clean up government complexity in the long run? How about the other option of simplifying the regulatory infrastructure instead and moving away from top down regulations to more responsive regulations? There are innovations happening in regulatory approaches which would be worth mentioning in your discussion. Please take a look at this paper – its something I’ve been involved in which might provide regulatory alternatives for LMICs (only in the distant future though): Porter *et al.* (2020 ^1^).
- And lastly as this work involves several project partners in Kenya it would be great to see one or more Kenyan co-authors. I’m sure their reviews and feedback will help to strengthen and validate your study findings and implications.

Is the work clearly and accurately presented and does it cite the current literature?

Partly

If applicable, is the statistical analysis and its interpretation appropriate?

Not applicable

Are all the source data underlying the results available to ensure full reproducibility?

No

Is the study design appropriate and is the work technically sound?

No

Are the conclusions drawn adequately supported by the results?

No

Are sufficient details of methods and analysis provided to allow replication by others?

Partly

Reviewer Expertise:

Health policy, health systems, private sector, qualitative research

I confirm that I have read this submission and believe that I have an appropriate level of expertise to confirm that it is of an acceptable scientific standard, however I have significant reservations, as outlined above.

## References

[1] : Using 'smart regulation' to tackle antimicrobial resistance in low-income and middle-income countries. *BMJ Glob Health* .2020;5(1) : 10.1136/bmjgh-2019-001864 e001864 10.1136/bmjgh-2019-001864 32133168PMC7042594

# Response

**Published:** 2021-10-04

**Authors:** Lauren Suchman

We would like to thank the reviewer for such thoughtful and useful comments. We have responded to comments point-by-point below.

  

- *The literature review is interesting and comprehensive but too discursive and it leaves little room for the findings.*
  - We have edited the literature review so that it is shorter, and more focused and relevant to the topics explored in the rest of the paper.
- *The analysis could have a lot more depth given that it is based on 126 semi-structure interviews and several FGDs. However I did not a convincing or cogent story emerging in the paper. The study aim is to show how the intermediary organisation addresses the barriers due to low level government workers’ issues but the findings have not fully engaged with or developed this theme. Partly this is because none of these street level bureaucrats (SLBs) have been interviewed, but even so, you could build a much more coherent story based on the responses of the recipient providers and the intermediaries. Tell us more about who these street bureaucrats are, how do they operate, what is the range of interactions that providers have with them, which interactions are the problematic ones, are there any issues that are commonly misinterpreted by the SLBs or are their actions random and arbitrary, when are bribes needed to be given, and what are the different tools/strategies through which the intermediaries/intervention deal with the different types of problems created by the laws and the SLBs. Is this a default outcome of the intervention or is it by design? Were there any differences in the way that the different provider types interacted with the SLBs?*
  - We have included more information in the Results section that we hope offers a more robust depiction of the street-level bureaucrats themselves, the obstacles they face to doing their job well, and the various ways in which they interact with providers. We hope this helps to create a more coherent story about the bureaucrats themselves, as well as the role that intermediaries can play in a complex regulatory environment.  
- *I feel that it would really help you to have a conceptual framework based on Lipsky’s theory, for your analysis to make greater sense of SLB behaviours and how the intervention helps providers deal with these behaviours.*
  - We certainly take and agree with your point. We removed this conceptual framework after a reviewer on an earlier draft suggested that it wasn’t quite working, but have added it back in at the end of the literature review and hope that it helps to bring the paper together.  

More detailed comments below:

 Results:

- *It would be very useful to begin with a description of the key actors involved in the scenario – the SLBs as well as the providers. You do tell us about the providers in the table - this could be summarised in the text drawing attention to the major provider types and the greater proportion of nurses and auxiliaries in the sample. Did you find any difference in the way that doctors coped with the regulations compared with nurses? If you found any differences, these could be unpacked and explained in the discussion.*
  - We have included more description of both the providers and street-level bureaucrats, as well as the franchise representatives to better help contextualize the results. Unfortunately, because the number of doctors interviewed was so low, it is difficult to tell if there is much difference in experience between those with this qualification and the nurses that made up the majority of the sample. Based on data we have published elsewhere (Suchman et al. (2020) Bridging the gap with a gender lens: How two implementation research datasets were repurposed to inform health policy reform in Kenya. Health Policy and Planning 35(S2): ii66–ii73.), we suspect there may be gender differences in the ways providers are able to navigate NHIF systems; this may align with qualifications since women are more likely to be nurses than doctors. However, we are currently pursuing funding to gather more data around this question. 
- *The findings are presented as very generalised statements rather than as qualitative evidence. For example: ‘Transparency and consistency effect the behavior of both the regulators and the regulated, creating or reducing loopholes, opportunities for corruption, and guidance on what one “should” do. Lack of transparency and consistency become both a barrier to effectiveness and an opportunity for front-line government workers to take shortcuts through the institutional red tape as a means to better carry out the mandates of their jobs. Can you present more direct evidence from the narratives that makes you arrive at this interpretation? Like (just a hypothetical example): ...several providers shared their experiences of having faced problems with SLBs like having their application forms rejected for accreditation for no apparent reason and not being available when approached for a clarification…(give a quote here...). This is especially important when you present evidence about corruption. It is not at all clear how the intervention has reduced or stopped this or made the path more ‘transparent and consistent’.*
  - We think that we have addressed this concern through our responses to other comments above re: bringing more detail and specificity into the analysis. We also have paid special attention to this issue while editing the results and have tried to cite direct evidence more often and include more illustrative quotes.

Methods:

- *It would be very useful to have a short description of the context/setting and the interventions upfront as these are critical to the study/analysis and the reader should understand them clearly to make sense of the findings.*
  - These points are already covered at the end of the Introduction under the sub-headings “The Kenyan context” and “The African Health Markets for Equity (AHME) NHIF accreditation assistance intervention.” We believe these sections as they are should be enough to frame the rest of the paper.  
- *What was the sampling universe? What was the total number of facilities of different types from where the sample was drawn?*
  - We have added some additional information to indicate that the franchise networks provided lists of recently franchised (i.e. franchised or approached to join the franchise during the period of the AHME program) facilities with each round of data collection. From these lists we selected facilities to participate in the study aiming for a distribution of providers across geography and range of experience with the AHME interventions.
- *It would be useful to have all the information about the tools and the piloting brought together under a sub-section on tools.*
  - We have re-organized the Methods section a bit so that it includes a “Tools” sub-section and the “Data collection” and “Data analysis” sub-sections are separated.
- *Please avoid long complex sentences like this one: "Upon arriving at the interview site, interviewers confirmed that the interviewee was one of the key people at the facility who had been involved in decision-making around whether or not to participate in the AHME interventions and obtained informed consent from the providers prior to conducting semi-structured interviews that lasted approximately 60 minutes each."*
  - We have broken this sentence into two sentences and also paid particular attention to sentence complexity while editing the rest of the paper.

Discussion:

- *I find that this section just repeats part of the introduction and the findings, without engaging with the findings. What would you say are your most important findings, what is their novelty, and how do you explain them? You need to go into greater depth to explain why intermediaries have been useful and I think this is why having a conceptual framework for this study would be very useful.*
  - We have re-written the Discussion to better address this point and think that it now draws more concretely on key study findings and their implications.
- *I would also have questions about the implications of your findings. How sustainable is it to have intermediary organisations help clean up government complexity in the long run? How about the other option of simplifying the regulatory infrastructure instead and moving away from top down regulations to more responsive regulations? There are innovations happening in regulatory approaches which would be worth mentioning in your discussion. Please take a look at this paper – its something I’ve been involved in which might provide regulatory alternatives for LMICs (only in the distant future though): Porter et al. (2020 1).*
  - Thank you for recommending this interesting and relevant article. We have incorporated your point and this citation into the Discussion and also edited the Discussion to include implications of the findings and recommendations for moving forward.
- *And lastly as this work involves several project partners in Kenya it would be great to see one or more Kenyan co-authors. I’m sure their reviews and feedback will help to strengthen and validate your study findings and implications.*
  - This point is well taken and while we had considered inviting a Kenyan colleague to join the paper earlier, we ultimately forged ahead and the paper clearly suffered as a result. Our esteemed colleague, Edward Owino, has since agreed to be a co-author. Mr. Owino is a health economist with extensive expertise in health financing and especially Kenya’s NHIF, and worked with the AHME program in this capacity.

# Peer review: Reviewer response for version 1

**DOI:** 10.21956/gatesopenres.14556.r30804

**Published:** 2021-06-28

**Authors:** Stefan Nachuk

**Affiliations:** 1 Bill & Melinda Gates Foundation, New Delhi, Delhi, India

This is an interesting and useful contribution to the field based upon a long-term intervention that aimed, in part, to bring private providers under the "purchasing umbrella" of the national health insurance fund in Kenya. Notwithstanding the qualitative nature of this work, there are a few opportunities to tighten the description of the larger evidence base generated by this project, and thus frame the findings within that larger effort. 

1. While the authors refer to "decentralization" in Kenya, this term is so broad that it is difficult to understand what it means in this context. A short summary of the extent to which Kenya decentralized administrative, political, and fiscal authority to lower levels would help frame this research somewhat better.  
2. Related to this, there is a rich body of work on decentralization and its impacts across the world. To simplify - two extreme positions are: a) it brings decision-making and accountability closer to the client (thus a good thing) and we should expect to see some positive results vs. b) it essentially creates more layers of corruption and gives unqualified bureaucrats more power. It's unclear if the authors had any particular expectations of what might happen in Kenya, and while there are some signals that clinic owners see positive signals, it is unclear if this is attributed to improved or more motivated NHIF functions, or to the positive effects of decentralization (or respondents did not venture any opinions on the subject).  
3. Since this is part of a much larger donor-funded project, it would be helpful to mention how many clinics were touched by the project in Kenya during its lifespan, and cite any information regarding differences in accreditation rates between those who had support vs. those that did not (if there were any that did not).  
4. The qualitative nature of this effort would lend itself to providing a bit more detail regarding what types of facilitative support were seen as particularly helpful by clinic owners vs. those that were only "nice to have" but did not do much for them. In addition, any thoughts they had regarding why this was the case would really help this piece. 
5. Finally, I think everyone would be interested to have some sense of the cost of replicating an effort like this within the Kenyan context. While a donor-funded effort is not the best baseline (i.e. it's expensive) it would still be useful to have some sense of what this support cost per clinic over time.  

It's a good and useful piece that gets to the heart of one of the binding constraints of moving towards UHC in many LMICs. Offering up a bit more context and detail would really help the findings jump out.

Is the work clearly and accurately presented and does it cite the current literature?

Partly

If applicable, is the statistical analysis and its interpretation appropriate?

Not applicable

Are all the source data underlying the results available to ensure full reproducibility?

Yes

Is the study design appropriate and is the work technically sound?

Yes

Are the conclusions drawn adequately supported by the results?

Yes

Are sufficient details of methods and analysis provided to allow replication by others?

Yes

Reviewer Expertise:

Health system, the role of the private sector within health systems, strategic purchasing.

I confirm that I have read this submission and believe that I have an appropriate level of expertise to confirm that it is of an acceptable scientific standard.
